# Treatment of Diphtheria

**Published:** 1880-10

**Authors:** 


### TREATMENT OF DIPHTHERIA.

Privat docent Dr. Jacob Schuetz, of Prague, having within the last seventeen years met with such marked success in the treatment of diphtheria with a solution of bromine (bromi puri, kali bromici ana 0. 5, aq. dest. 100.0), feels prompted to call the attention of the physician again to this cardinal remedy. He employs the medicine in the form of a vapor inhalation when the air passages are involved, else as a gargle or an injection, or even pencils the diseased surfaces with it. The injections are to be preferred to the gargles, as in the process of gargling the tongue assumes such a position as to render thorough washing of the fauces almost impossible. Lime-water or a weak solution of bromine may answer for the injection and gargle, but the penciling is to be performed with the concentrated bromic solution. The author, after having ineffectually tried to remove the membranous layer with a pincette, successfully adopted the following very simple method: Wrapping with a fine, clean piece of linen the index finger of either the right or the left hand, as the site of the disease may indicate, then dipping it into water, he rubs over the part, which on examination he has found covered with a membranous layer. Almost invariably more or less of this, clinging to the linen, will become detached. In many cases the layer, having been rendered loose by the procedure, is expelled subsequently by the efforts of hawking which the irritation induces, or by the injections, which are practiced immediately after the friction. In this manner he was often able to remove large and numerous masses from the affected parts. To determine whether this deposit was diphtheritic or croupous in nature, he had these masses examined at the pathological anatomical institute of Prague. In twenty-eight cases the author had an opportunity of witnessing the efficacy of this method. The resistance with which it in the beginning met at the hands of the patient and his friends was easily overcome, while the progress of the disease after the first application of friction was really marvelous. Inspection revealed the parts to be either partially or entirely clear. From that moment on, the condition of the patient also improved. The symptoms of febrile movement that might have been present quickly evanesced, the appetite returned and refreshing sleep was again enjoyed. Though new formations were going on, the patients looked cheerful and happy. These new formations, however, did not display the severity of the first formed layer, and the parts from which the latter had been separated were, as a rule, spared. As the whole membranous deposit can only in exceptional instances be removed at once, it will be necessary to make frequent attempts at friction for a variable period of time. According to Schuetz there are some cases which may be pronounced cured in two or three days. The injections which are to be made after the detachment of the layer, can be practiced by adults themselves with a pear-shaped rubber syringe. The removal of every newly formed patch of membrane may be entrusted to some friend. "If," continues the author, "my view that diphtheria is a local disease be disputed, or be proven incorrect, the fact nevertheless stands incontestable that it is the membranous layer which spreads, and spreading may invade the air-passages and cause suffocation. Again, experience teaches us that once the air-passages are implicated, the morbid process has a tendency to extend downward into the bronchioles. In that case, tracheotomy even would be of little avail, since the membranous deposit reaches farther below than above the seat of the operation, and when torn out rapidly reproduces itself. Hence, under these circumstances, it may become a matter of some consequence to know whether the removal of the first formed layer would not limit its extension and thus avert all danger. Should this prove correct the mechanical friction recommended here, may be the best, if not the only means at your disposal. I am already forced strenuously to oppose the idea entertained at present, that all injury to the layer must be avoided, because it excites the formation of new membranous layers. In the cases in which friction was employed after the manner indicated, the injured and bleeding surfaces were not again affected, and Professor Klebs, to whom I showed one of the cases, can substantiate the fact. The happy results which this method of treatment secured, the slight pain it occasioned, and the general state of well-being it induced were so remarkable that two families, particularly obnoxious to this disease, employed it at the slightest indication, even before consulting my advice. The observation was also made by them, that the layer could be stripped off more easily when the bromine solution was used, than when lime water was simply applied. The method of treatment employed in the twenty-eight cases treated up to this time, and ranging between three and sixty years of age, may be thus summarily expressed:

1. As soon as any part of the fauces is covered with a membranous layer, this must be washed and rubbed off in the manner described. Should it not come off entirely, the process must be immediately repeated; 2. As soon as the fauces are clear, two or three injections with a weak solution of bromine or simply water must be made by means of the rubber syringe; 3. The rubbing off must be practiced two or three times daily until no trace of deposit is visible; 4. In the intervals, the injections, which the patient can make himself, are to be repeated hourly; 5. In employing bromine solution as an injection, it is advisable to begin with a weak solution, gradually increasing the strength; 6. Should any membranous patch resist the described procedure, it would be judicious to pencil it with the concentrated solution of bromine, five or six times a day; 7. I am convinced that the cold compresses around the neck which are popularly in such high repute are not at all essential; 8. Swelling of the glands about the neck and lower maxilla may be treated with an iodine salve; 9. I isolate my patient and after recovery request them, as a matter of precaution, to ventilate the bed-clothes, allowing them, however, to adopt any method of disinfection they may deem proper."—*Med. Neuigkeiten*, No. 23, 1880.—*Lancet and Clinic.*

